# Effects of COVID-19 on parental perception of children’s oral health-related quality of life

**DOI:** 10.1590/1807-3107bor-2026.vol40.015

**Published:** 2026-05-08

**Authors:** Thays Torres do Vale OLIVEIRA, Marília Leão GOETTEMS, Vanessa Polina Pereira COSTA, Fausto Medeiros MENDES, Thiago Machado ARDENGHI, Marina Sousa AZEVEDO

**Affiliations:** (a) Universidade Federal de Pelotas – UFPel. Graduate Program in Dentistry, Pelotas, RS, Brazil.; (b) Universidade de São Paulo – USP, School of Dentistry, Graduate Program in Dentistry, São Paulo, SP, Brazil.; (c) Universidade Federal de Santa Maria – UFSM, School of Dentistry, Graduate Program, Santa Maria, RS, Brazil.

**Keywords:** Coronavirus, Oral Health, Quality of Life, Child

## Abstract

The aim of this study was to evaluate the effects of the COVID-19 pandemic on parents’ perceptions of their children’s oral health related quality of life (OHRQoL). This longitudinal study was performed in two stages: baseline (T1), before the pandemic, and follow-up (T2), from August to November 2021. Children aged eight to eleven years and their parents who were seeking dental care or were referred to the Pediatric Dental Clinic of the School of Dentistry, residing in Pelotas, state of Rio Grande do Sul (RS) were included. OHRQoL served as the primary outcome measure and was assessed using the Brazilian short-form of the Parental-Caregiver Perceptions Questionnaire (P-CPQ) at T1 and T2. Child’s sex and age, caries status (dmft/DMFT), parents’ education, and household income were collected at T1. Variables related to COVID-19 were collected at T2: fear of COVID-19 and of family virus transmission, job loss, and children’s dental pain in the past 4 weeks. The non-parametric Wilcoxon test was used to compare OHRQoL. Changes in OHRQoL over time were analyzed using a multilevel negative binomial regression model. The random intercept and fixed effects model considered repeated measurements (level 1) grouped into individuals (level 2). The level of significance was set at 5%. There was no statistically significant difference between the median for total score and for each domain. In the multilevel analysis, fear of COVID-19 was linked to poorer OHRQoL perceptions. Having dental pain and younger children also negatively impacted these perceptions. Emotional and psychological factors and non-normative parameters significantly influenced parental perceptions of OHRQoL during the pandemic.

## Introduction

The SARS-CoV-2 pandemic spread to over 230 countries around the world by the end of May 2024.^1^ Causing different respiratory symptoms and deaths, the “Coronavirus Disease 2019 (COVID-19)” was classified by the World Health Organization as an international health emergency.^2^ In June 2020, Brazil was the second most affected country in the world by the pandemic, almost 1.5 million confirmed cases, surpassed only by the United States. Brazil finished 2021 with more than 619 thousand deaths caused by COVID-19, and April was the month with the highest number of confirmed deaths from COVID-19.^3^

Transmission of respiratory viruses occurs during exhalation, speech, and during coughing and sneezing. Therefore, rules of social distancing, use of face masks, hand hygiene, use of personal protective equipment, and stay-at-home orders were adopted in many countries to reduce the incidence of COVID-19.^4-7^ These transmission control measures considerably changed people’s lives, including those of children, whose school-based classes were suspended.^8,9^

In dental practice, the pandemic caused a reduction in routine care, disrupting preventive appointments and dental treatments.^10-13^ Economic issues, general concerns, fear, combined with the lack of preventive dental care and postponement of elective treatments, could impact children’s oral health during enforced stay-at-home orders.^14^ Therefore, concerns about children’s oral health-related quality of life (OHRQoL) have become a topic of discussion.^14-16^

OHRQoL is an important indicator of an individual’s satisfaction (or parents’ perception, in the case of children) with their oral health based on oral health conditions, general health, and social and environmental interactions.^17,18^ Given that OHRQoL can be affected by different factors, it is important to study the impact of the COVID-19 pandemic, with social and school isolation and economic problems, on the subjective aspects of parents’ perception of their children’s quality of life. Knorst et al.^16^ conducted a cohort study with adolescents from southern Brazil to evaluate the immediate effects of the COVID-19 pandemic on their OHRQoL. The results showed a decrease in the perception of oral health problems by adolescents in that period, indicating that the COVID-19 pandemic reduced their negative perception.^16^

To date, there has been no study in the literature evaluating the impact of the COVID-19 pandemic on the perception of parents of school-aged children in the 7-11 age group regarding their OHRQoL. Thus, the aim of this longitudinal study was to evaluate the effects of the COVID-19 pandemic on parent’s perceptions of their children’s OHRQoL.

## Methods

This study followed the Strengthening the Reporting of Observational Studies in Epidemiology (STROBE) guidelines.^19^

### Ethical issues

This project was approved by the Research Ethics Committee of the Federal University of Pelotas (UFPel) under no. 3.282.962. All participants were informed about the study objectives and signed the informed consent form from the original study.

### Study design and sampling

The study was conducted at the Pediatric Dental Clinic, School of Dentistry, UFPel, Pelotas, state of Rio Grande do Sul, Brazil. The sample was part of a larger study^20^ that was suspended in March 2020 due to the COVID-19 pandemic. Participants who were recruited for the larger study up until February 2020 were selected to participate in this longitudinal study.

We used the sample recruited prior to the pandemic. Due to the ready availability of the participants and their data, the authors considered this a convenience sample for the study. In this sense, the power calculation accounted for an alpha error probability of 0.05, P-CPQ scores of 1.37 (standard deviation [SD] 1.6) at T1 and 1.83 (SD 1.86) at T2, resulting in a sample power of 33%.

This longitudinal study was performed in two stages: baseline (T1) and follow-up (T2). At baseline, children aged eight to eleven years and their respective legal guardians who were seeking dental care or were referred to the Pediatric Dental Clinic of the School of Dentistry - UFPEL - residing in Pelotas/RS or nearby, were included. Children who wore fixed orthodontic appliances and who had systemic diseases or a disability that limited understanding of the questions that would be asked were excluded. T1 included 88 pairs of children-caregivers who were followed up. Subsequently, all these parents were contacted again (T2) to evaluate possible impacts of the pandemic on the perception of their children’s OHRQoL.

### Data collection

Baseline data collection (T1) was performed from July 2019 to February 2020. Data were collected by trained interviewers at the Pediatric Dental Clinic. Subsequently, from August to November 2021, the previously assessed parents were contacted by telephone for a new data collection (T2). The phone calls were made by a trained interviewer. Three call attempts were made, in different shifts, through conventional calls and also three shifts on different days through WhatsApp® messages (Meta Platforms Inc.).

Demographic, socioeconomic, and behavioral characteristics and OHRQoL were assessed in both evaluations. At T1 and T2, OHRQoL was assessed using the Brazilian short form of the Parental-Caregiver Perceptions Questionnaire (P-CPQ)^21^ with the same respondent answering the questionnaire in both periods. The short-form P-CPQ has a total of 13 questions organized as follows: have three questions about oral symptoms, with a minimum score of zero and a maximum of 12; four questions on the functional limitations domain, with a minimum score of zero and a maximum of 16; and six questions about the well-being domain, with a minimum score of zero and a maximum of 24. The minimum score for the entire questionnaire was zero and the maximum was 52 points. The higher the score, the worse the OHRQoL.

Data on monthly household income, child’s age and sex, and parent’s educational level were collected using a semi-structured questionnaire. Household income was recorded in Brazilian currency (Reais), converted to US dollars, and dichotomized into ≤ $353 and > $354 according to the median. Child’s age was recorded in years and dichotomized into 7– and 10–11 years, and the mean age of the sample was 9.6. Parents’ educational level was recorded in years of schooling and dichotomized into up to 8 years and more than 8 years.

At baseline, a calibrated single examiner conducted the children’s oral examinations using the decayed, missed or filled tooth (dmft/DMFT) index,^22^ with a appa of 0.95 for dmft/DMFT. The mean dental caries status was used to analyze the results.

Some variables related to the COVID-19 pandemic were collected at T2. A question related to COVID-19 contamination was assessed: “*Have you and/or anyone else at home had COVID-19*?,” dichotomized as ‘no’ and ‘yes’. A question related to job loss was assessed: “*Has anyone in the family lost their job or been economically harmed due to the pandemic?*,” the response options were: ‘No’, ‘Yes and lost job,’ and ‘Yes, economically affected’, later dichotomized as ‘no’ or ‘yes’. Regarding the fear of COVID-19, the following statement was presented: ‘I am very afraid of COVID-19.’ Respondents had the following response options: ‘strongly disagree,’ ‘disagree,’ ‘neither agree nor disagree,’ ‘agree,’ and ‘strongly agree’.^23^ Responses of ‘agree’ and ‘strongly agree’ were categorized as ‘yes,’ while all other responses were categorized as ‘no’.

Dental pain was also investigated with the question: “*Has your son/daughter had dental pain in the past 4 weeks?*”.^24^ The answer options for dental pain were ‘ ‘no’ and ‘yes’. Parents were asked whether their children had visited a dentist during the period from March 2020 (the onset of the pandemic) to T2.

### Statistical analysis

Data analyses were performed using STATA 17.0 (Stata Corporation, College Station, USA). A descriptive analysis of the sample was carried out according to the characteristics evaluated at T1 and T2. The comparison between individuals from T1 and dropouts was assessed using the chi-square test. The Kolmogorov-Smirnov test was used to test the normality of the outcome, and the assumption of normally distributed data was rejected. The non-parametric Wilcoxon test was used to compare OHRQOL from before (T1) and after the onset of the COVID-19 pandemic (T2). Changes in quality of life over time were analyzed using a multilevel negative binomial regression model. The random intercept and fixed effects model considered repeated measurements (level 1) grouped into individuals (level 2). Variables that presented a p value of up to 0.20 in the unadjusted analysis were considered in the adjusted model. In this final model, only variables with a p value of up to 0.05 were maintained. The level of significance was set at 5%.

## Results

From 88 individuals evaluated at T1, 59 were re-evaluated at T2 (response rate of 67%). The main reason for loss to follow-up was due to the inability to contact the participants by phone (n = 28), and one participant refused to participate. Regarding the followed-up participants and non-respondents’ caregivers, there was no difference regarding sex (p = 0.49), age (0.59), household income, (p = 0.32), parents’ education (p = 0.14), and untreated dental caries (p = 0.70).

Table 1 shows the characteristics of individuals who were followed up at T2. Characteristics collected before (baseline) and after the onset of the COVID-19 pandemic in Brazil (follow-up) are presented. Among the sampled individuals, 50.8% were female, most of the children were aged 10 to 11 years (54.2%), 27.1% reported dental pain in the past 4 weeks, and the mean dmft/DMFT score was 2.17 (SD 2.23). Regarding socioeconomic characteristics, most of the parents had up to 8 years of formal education (50.8%). According to information obtained at T2, 81.4% of the families were afraid of COVID-19, 64.4% of participants did not have COVID-19, and 61% lost their job or were economically affected in some way. From the total of re-evaluated children , 5% had sought dental care from the onset of the pandemic to the time when the questionnaire was applied at T2.

**Table 1:** Sample characteristics before and after the onset of the COVID-19 pandemic in Brazil. Pelotas, RS (n = 59).

Variables	n	%
Baseline (T1) characteristics		
Children’s sex		
Female	30	50.8
Male	29	49.2
Children’s age (years)		
7–9	27	45.8
10–11	32	54.2
Parents’ education (years)		
0–8	30	50.8
≥ 9	29	49.2
Household income (median)		
≤ U$353	29	49.2
> U$354	30	50.8
Follow-up (T2) characteristics		
Loss of job/economically affected		
No	23	39.0
Yes	36	61.0
Family had COVID-19		
No	38	64.4
Yes	21	35.6
Fear of COVID-19		
No	11	18.6
Yes	48	81.4
Children dmft/DMFT		
mean (SD )	2.17	2.23
Dental pain in the past 4 weeks		
No	43	72.9
Yes	16	27.1

Overall and domain-specific P-CPQ scores and changes between baseline and follow-up are presented in Table 2. Except for the functional limitation domain, all other domains and the total P-CPQ score were higher after the onset of the COVID-19 pandemic, and no statistically significant difference was observed. Regarding total scores, the overall P-CPQ was 5.63 (SD 5.61) at T1 and 6.00 (6.08) at T2, with a difference of 0.37 in the means . The largest difference was observed in the oral symptoms domain, which was 0.46 higher from T1 to T2. There was no statistically significant difference between the median for the total score and for each domain comparing the baseline (before COVID pandemic) and follow-up (after the onset of the COVID pandemic).

**Table 2:** Changes in overall and domain-specific P-CPQ scores in the sample before and after the onset of the COVID-19 pandemic in Brazil.

Outcome (P-CPQ)	Baseline (T1)			Follow-up (T2)			Difference between means (T2-T1)	p-value*
	Mean (SD)	Median	(IQR)	Mean (SD)	Median	(IQR)		
Oral symptoms	1.37 (1.60)	1	(0–2)	1.83 (1.86)	2	(0–2)	0.46	0.106
Functional limitations	2.27 (2.45)	2	(0–4)	2.10 (2.68)	2	(0–3)	-0.17	0.371
Emotional well-being	1.42 (2.48)	0	(0–2)	1.49 (2.18)	0	(0–2)	0.07	0.663
Social well-being	0.56 (1.65)	0	(0–0)	0.58 (1.62)	0	(0–0)	0.02	0.934
Total score	5.63 (5.61)	4	(2–7)	6.00 (6.08)	4	(2–8)	0.37	0.907

SD: standard deviation; IQR: interquartile range. *Wilcoxon test.

Table 3 demonstrates the changes in the overall P-CPQ scores during the COVID-19 pandemic according to sociodemographic and clinical variables. In the univariate analysis, the variables “Children’s mean dmft/DMFT”, “children’s age”, “fear of COVID-19,” and “dental pain in the past 4 weeks” were associated with changes in OHRQoL. After adjustments, the variables “children’s age,” “fear of COVID-19,” and “dental pain in the past 4 weeks” remained significant. Younger children, whose parents were afraid of COVID-19, and who had a toothache in the past 4 weeks had a worse quality of life when compared to their counterparts (Table 3).

**Table 3:** Changes in overall P-CPQ scores during the COVID-19 pandemic in Brazil according to household income, level of education, age, employment status, history of COVID, fear of COVID, dental caries, and dental pain determined by multilevel Poisson regression model for repeated measures.

Variables	P-CPQ total score			
	Baseline	Follow-up	Unadjusted	Adjusted
	Mean (SD)	Mean (SD)	Mean (SD)	Mean (SD)
Children’s age (years)				
10–11	4.87 (4.70)	4.28 (4.22)	1	1
7–11	6.52 (6.50)	8.04 (7.30)	1.54 (1.04–2.28)	1.43 (1.01–2.02)
Household income (median)				
> U$354	5.50 (5.54)	5.37 (5.48)	1	
≤ U$353	5.76 (5.78)	6.65 (6.68)	1.16 (0.78–1.75)	
Parents’ education (years				
0–8	6.38 (6.93)	6.79 (6.29)	1	
≥ 9	4.9 (3.92)	5.23 (5.88)	0.82 (0.54–1.24)	
Job loss/economically affected				
No	5.91 (5.55)	6.56 (7.00)	1	
Yes	5.44 (5.71)	5.64 (5.49)	0.88 (0.58–1.34)	
Family had COVID-19				
Yes	6.19 (6.11)	7.24 (7.25)	1	
No	5.31 (5.32)	5.31 (5.31)	0.80 (0.52–1.21)	
Fear of COVID-19				
No	3.00 (2.57)	2.73 (3.50)	1	
Yes	6.23 (5.95)	6.75 (6.32)	2.26 (1.34–3.83)	1.91 (1.19–3.08)
Children’s dmft/DMFT				
mean			1.11 (1.01–1.21)	
Dental pain in the past 4 weeks				
No	4.60 (4.76)	4.32 (4.16)	1	1
Yes	8.37 (6.86)	10.5 (8.07)	2.18 (1.45–3.27)	1.84 (1.26–2.70)

## Discussion

The findings of this study highlighted that, although there was an overall increase in P-CPQ scores across most domains, these changes were not statistically significant when pre- and post-pandemic periods were compared . However, the adjusted multilevel analysis revealed that younger age, parental fear of COVID-19, and dental pain in the past 4 weeks were significant predictors of worse OHRQoL, as perceived by parents.

The fear of COVID-19, called ‘coronaphobia,’ caused by the uncertainty and unpredictable nature of the disease and fear of being infected, leads to mental distress and behavioral change in people.^25^ Also, the lockdown measures affected people’s mental health with several psychological consequences.^26^ The fear of COVID-19, in particular, can exacerbate concerns about the overall health and well-being of their children, causing parents to perceive a deterioration in their children’s OHRQoL, even in the absence of objective changes. This study reinforces the importance of considering psychological factors and the emotional context of parents when evaluating perceptions related to their children’s health.

The findings of this study indicated that parents of children who reported children’s dental pain in the past 4 weeks during the pandemic perceived worse quality of life. The pandemic severely affected healthcare services, including dental care, resulting in disruptions and limitations in access to essential treatments and preventive services.^27^ A study showed that the pandemic significantly affected the number of pediatric dental procedures performed in primary health care in Brazil, leaving several school-aged children without dental care during the COVID-19 pandemic.^28^

Also, many parents have delayed seeking the necessary care for their children. In a Brazilian study with parents of children aged 0–12 years during the pandemic, almost 40% did not seek dental treatment when their children experienced dental pain.^14^ Thus, it is suggested that the lack of dental care may have made children more vulnerable to oral problems, mainly due to their inability to solve their dental problems, generating pain, and may thus increase the impact of these oral conditions on OHRQoL.

A cross-sectional study was conducted among Indian parent-child dyads to investigate the impact of a child’s dental pain, caregiver’s fear of SARS-CoV-2, and parental distress on the oral health-related quality of life (OHRQoL) of preschoolers during the COVID-19 pandemic lockdown. The survey results revealed that caregivers experiencing higher levels of stress and fear of COVID-19, along with children reporting more severe dental pain, had the lowest quality of life scores during the pandemic,^29^ consistent with the findings of this research. The authors also hypothesized that increased fear of COVID-19 among parents may have affected their decision not to seek dental care during the pandemic, potentially exacerbating unmet dental needs in their children.

Furthermore, in this Indian study, a dmft score greater than 5 significantly increased the risk of poor OHRQoL for the child.^29^ Contrary to this finding, our study did not reveal a deterioration in OHRQoL when assessing the mean dmft/DMFT. In our study, there was no perceived decline in OHRQoL associated with the mean DMFT. The authors hypothesize that this lack of significant change in dmft/DMFT may be attributed to the inclusion of dental pain and dmft/DMFT in the same statistical model.

Considering that the primary cause of dental pain in this age group is untreated caries (decayed component), it is believed that dental pain persisted due to its greater impact. A two-point increase in the mean score was observed among children whose parents reported dental pain in the past 4 weeks between baseline and T2, a trend that was also noticeable in the oral symptoms domain (data not shown). Children who already had experienced dental caries, with decayed teeth, are more vulnerable to the deterioration of their oral health status and, consequently, to dental pain, combined with the decrease in access to dental services during the pandemic.

Of note, other oral health conditions, such as dental trauma, can also have a significant impact on OHRQOL. Although dental pain can encompass a range of conditions, including dental trauma^30^ and some oral mucosal lesions, when interpreting the findings, it is important to consider this study did not assess diseases other than dental caries.

The findings of this study revealed that during the COVID-19 pandemic, parents perceived a worse OHRQoL for younger children (aged 7–9 years) compared to older children (those aged 10–11 years). This disparity may be attributed to several factors. Younger children typically require more parental assistance for oral hygiene practices, are less capable of articulating or managing dental pain and this further complicates access to effective dental care, which could increase parental anxiety and perception of poor OHRQoL during a period marked by restricted access to dental care services.^28^ Thus, the disruptions in routine dental visits caused by the pandemic may have particularly affected younger children. In addition to these difficulties, younger children exhibited a higher burden of dental caries disease, primarily from the decayed component.^31^ This increased burden was also observed in our sample (data not shown). A study during the COVID-19 pandemic in Kwait showed that OHRQoL scores decreased with age.^32^ A cohort study in Brazil that evaluated the immediate effects of the COVID-19 pandemic on OHRQoL among schoolchildren did not identify age-related differences; however, the sample in that study consisted of children aged 10–15 years.^16^

In the same study, the overall OHRQoL scores showed a decrease, indicating a reduction in the perception of oral health problems during this period,^16^ which differs from the findings of our research. A possible explanation for this difference in results may be related to the data collection period, as the mentioned research was conducted right at the beginning of the pandemic, a time when other concerns such as the economy and fear of contracting the virus were more prominent.^14^ In addition to oral health not being the main concern during this initial period of the pandemic, the short timeframe likely meant that unmet oral health needs had not yet accumulated to the point of significantly impacting OHRQoL.

It is important to highlight the implications of these findings for both clinical practice and public health. Clinicians should adopt a holistic approach when assessing children’s OHRQoL, considering not only clinical (normative) measures, but also emotional and psychological factors. Understanding the emotional state of parents can provide an important context for tailoring treatment plans. Furthermore, public health initiatives should emphasize the importance of maintaining oral health during pandemics and other public health crises. Policies and resources should be directed towards ensuring that families, especially those most affected by the public health crises, have access to both dental and mental health services. This integrated approach is crucial for mitigating the adverse effects of public crises, as we have had during the COVID-19 pandemic, on children’s oral health and overall well-being.

A significant limitation of this study was its reduced sample size, which was originally intended to be larger as part of a broader study that had to be suspended in March 2020 due to the COVID-19 pandemic. The interruption of participant recruitment before reaching the planned sample size resulted in a convenience sample, potentially limiting the generalizability of the findings. Additionally, due to the pandemic, a small sample size was expected. This may have impacted our results, mainly due the low power of the statistical test. Therefore, the results presented here should be interpreted with caution. However, our findings help to understand the impact of COVID-19 on families and children’s oral health, highlighting the need for more prospective studies to establish causality. Although only one collection was made during the pandemic, this study makes a notable contribution to the existing literature by addressing a significant gap, exploring the impact of the pandemic on parental perceptions of their children’s OHRQoL — an area that has remained largely unexplored so far. It is important to consider that data collection at follow-up (T2) was conducted before the start of vaccination in Brazil. Data collected after the beginning of vaccination or even after the declaration of the end of the pandemic might yield different findings from those observed in this study.

Additionally, our study included a comprehensive comparison between participants who completed the follow-up assessments and those who did not. Our analysis did not reveal any significant differences, suggesting minimal risk of non-response bias affecting the interpretation of our findings.

## Conclusion

In conclusion, our results suggest that emotional and psychological factors are important considerations when assessing parental perceptions of OHRQoL, as evidenced by the finding that fear of COVID-19 led to a poorer perception of OHRQoL. The total P-CPQ score did not show a statistically significant difference, although it had presented higher values after the beginning of the pandemic, with a difference of 0.37.

Additionally, dental pain emerged as a significant factor negatively influencing parental perception. Furthermore, parents of younger children also reported a worse perception of OHRQoL. These findings underscore the need for comprehensive assessments that encompass emotional and non-normative aspects alongside clinical measures for evaluating children’s OHRQoL during the COVID-19 pandemic.

## Data Availability

The datasets generated during and/or analyzed during the current study are available from the corresponding author on reasonable request.
